# Effects of limonin treatment on the survival of random skin flaps in mice

**DOI:** 10.3389/fsurg.2022.1043239

**Published:** 2023-01-06

**Authors:** Ting Zhang, Qing Huang, Kaifeng Gan, Ke Zhou, Keqi Hu, Wei Ding, Jiale Jin, Jin Li

**Affiliations:** ^1^ Department of Orthopaedics, Li Huili Hospital Affiliated to Ningbo University, Ningbo, China; ^2^ The Second School of Medicine, Wenzhou Medical University, Wenzhou, China

**Keywords:** limonin, random skin flap, angiogenesis, oxidative stress, apoptosis

## Abstract

Random skin flap is commonly used in plastic and reconstructive surgery, however, distal part of skin flap often occurs ischemia and necrosis. Limonin, with bioactivities of anti-inflammation, anti-apoptosis and anti-oxidative stress, may be effective for skin flap survival. In our study, random flap model was performed in mice to explore the role of limonin in the survival of skin flap. On postoperative day 7, the necrosis of skin flaps was observed, while visualization of blood flow below the tissue surface was detected through Laser Doppler blood flow imaging (LDBFI). Then flap tissues were acquired to assess and levels of angiogenesis, apoptosis and oxidative stress. The results showed that limonin decreased necrosis and edema of skin flaps compared with the control group, with more blood flow in the flap under LDBFI detection. Limonin treatment also increased the mean vessels density, elevated the expression levels of angiogenic proteins (matrix metallopeptidase 9, vascular endothelial growth factor, Cadherin5) and antioxidant proteins [superoxide dismutase 1 (SOD1), endothelial nitric oxide synthase, heme oxygenase], and reduced the expression of apoptotic factors (BAX, CYC, Caspase3). In summary, limonin could effectively enhance the survival of random skin flap, the potential mechanism may attribute to the induction of angiogenesis, and inhibition of apoptosis and oxidative stress.

## Key messages

• Strategies for improving the vitality of random skin flaps and inhibiting the necrosis related factors are critical for clinical application.
• The aim of our study is to explore the therapeutic actions of limonin in skin ﬂaps.
• Limonin is effective for the survival of random skin flap, through induction of angiogenesis, and inhibition of apoptosis and oxidative stress.

## Introduction

Random-pattern skin flap grafting is commonly used during plastic and reconstructive surgery (1). It results in flexibility of the skin flap due to no limitation of specific blood vessels, and the microvascular network at the junction of the skin flap and tissue usually promotes the survival of the skin flap through nutrition support (2, 3). Thus, it is effective to repair and reconstruct the deep skin damage and relevant soft tissue damages, which may occur in the trauma and surgical procedures. However, insufficient blood supply at distal flaps is prone to develop a vulnerability of tissue ischemia and necrosis, accompanied by inflammatory responses and strong oxidative stress damages (4, 5). Therefore, seeking novel approaches to improve the vitality of random skin flaps and inhibit the necrosis related factors are critical for clinical application of random flaps.

Limonin, a natural tetracyclic triterpenoid compound originally extracted from citrus seeds, is usually considered as one of the medical and edible botanicals with a unique taste and riches in vitamins (6, 7). Previous studies have indicated that limonin exhibited various pharmacological effects, including anticancer, antifungal, anti-inflammatory, antioxidant, and analgesic effects, which involved in the regulation of cancers, inflammatory diseases and metabolic diseases (8–10). As reported, limonin was able to alleviate hepatocellular injury after liver ischemia and reperfusion, and the mechanism of these hepatoprotective effects may attribute to the antioxidant and anti-inflammatory potential of limonin through toll-like receptor dependent pathway (11). In addition, it has also been shown that limonin inhibited IL-1β-induced inflammation and catabolism responses in chondrocytes and attenuated the development of osteoarthritis, indicating its significant effect of anti-inflammation (12). Considering the potent effect of limonin on anti-inflammatory and antioxidant, and ischemia related diseases, we wonder that limonin may protect against ischemia and necrosis on random skin flaps.

In this study, we comprehensively investigated the of limonin on random skin flaps survival, as well as its potential effect on angiogenesis and antioxidant stress, exploring the therapeutic actions of limonin in skin ﬂaps.

## Material and methods

### Animals

Adult C57BL/6J mice (8–12 weeks, Shanghai SLAC Laboratory Animal Corporation, China) were used in this study. All animal experiments were conducted strictly followed the Guide for the Care and Use of Laboratory Animals of the China National Institutes of Health. All procedures involving mice were carried out with approval of The Tab of Animal Experimental Ethical Inspection of the XXXX. All mice in this experiment were housed individually in standard experimental cages, with a 12-h light/dark cycle and free access to sufficient food and water before any experimental procedure. Thirty-six mice were used and randomly divided into the control and limonin-treatment groups (*n* = 18 for each group).

### Reagents and antibodies

Limonin (C_32_H_42_O_14_, purity ≥98%) was purchased from Solarbio (Beijing, China). Diaminobenzidine (DAB) developer, pentobarbital sodium, and the hematoxylin and eosin (H&E) staining kit were provided by Solarbio (Beijing, China). Rabbit anti–cadherin 5 was purchased from Boster Biological Technology (A02632–2). Rabbit anti-GAPDH was acquired from Biogot Technology (AP0063). Rabbit anti–vascular endothelial growth factor (VEGF), anti–superoxide dismutase 1 (SOD1), anti–matrix metalloproteinase 9 (MMP9), anti-HO-1, and anti-CAPS3 were acquired from Proteintech (19003-1, 10269-1, 10375-2, 21327-1, and 19677-1). Rabbit anti–endothelial nitric oxide synthase (eNOS), anti–cytochrome c (CYC), and anti-Bax were purchased from Cell Signaling Technology (12994, 14796, and 32027). Horseradish peroxidase (HRP)–conjugated immunoglobulin G (IgG) secondary antibody was purchased from Santa Cruz Biotechnology. Fluorescein isothiocyanate (FITC)–conjugated IgG secondary antibody was obtained from Boyun Biotechnology, and the 4′,6-diamidino- 2-phenylindole (DAPI) solution was purchased from Beyotime Biotechnology. The Electrochemiluminescence (ECL) Plus Reagent Kit was obtained from PerkinElmer Life Sciences and the BCA kit was acquired from TermoFisher Scientifc.

### Random-pattern skin flap model

Before surgery, mice were anesthetized by intraperitoneal injection of 50 mg/kg 1% (w/v) pentobarbital sodium. Then a caudal-based skin/panniculus carnosus ﬂap (size 1.5 × 4.5 cm^2^) was elevated on the mouse dorsum beneath the fascia using a mouse dorsal random ﬂap model as previously reported (13). After that, the right and left sacral arteries supporting the blood supply of this ﬂap were excised completely. Finally, the separated ﬂap was inset immediately into the donor bed and sutured using 5–0 silk. The random ﬂap area was divided into the proximal (area I), intermediate (area II), and distal (area III) zones, each with the same size. On day 7 post surgery, all mice were euthanized with an overdose of pentobarbital sodium. Six mice in each group were sacrificed for Western blotting analysis, and six mice in each group sacrificed for immunohistochemistry (IHC) analysis and H&E staining, while six mice in each group were used for the general evaluation of survival, the tissue edema assessment, and laser Doppler blood ﬂow imaging.

### Drug administration

Limonin was dissolved in 1% dimethyl sulfoxide (DMSO)–saline solution to a concentration of 50 mg/ml. Each mouse in the limonin group received limonin (40 mg/kg per day) by intraperitoneal injection for 7 days after the surgery. Mice in the control group received equal volume of 1% DMSO–saline solution (vehicle control) for 7 days. At the day 7 after the surgery, all mice were euthanized by pentobarbital sodium (overdose), and skin samples were harvested.

### General evaluation of flap survival

Macroscopic development and characteristics of texture, appearance, color, and hair condition of the ﬂaps were observed for 7 days after the surgery. At 3 and 7 days after the surgery, images of the skin ﬂap were acquired to estimate ﬂap viability, and survival area were measured using Image-Pro Plus imaging software (version 6.0; Media Cybernetics, Silver Spring, MD, United States), the percentage of viable area was measured as follows: the extent of viable area × 100%/total area.

### Assessment of tissue edema

Tissue edema is a crucial indicator of necrosis in ischemic ﬂaps. Tissue edema was determined according to the water content of the ﬂaps. On postoperative day 7, six ﬂap tissue samples from each group were weighed, dehydrated in an autoclave at 50 °C, and weighed until weight remained stable for at least 2 days. The percentage of water was measured as [(wet weight − dry weight)/wet weight] × 100%.

### Laser Doppler blood flow imaging

The blood supply and vascular ﬂow within the ﬂaps were evaluated by laser Doppler blood ﬂow (LDBF) measurements (14). On postoperative day 7, a laser Doppler instrument (Moor Instruments, Axminster, UK) was applied to scan the dorsal skin of mice in a safe environment under anesthesia. LDBF evaluation was performed according to the previous study (15). The entire dorsal skin of mice, including the ﬂap area, was scanned by laser Doppler. Laser Doppler blood ﬂow commonly provides deeper penetration, which enables reinforced visualization of small vessels under the tissue surface, which is suitable for angiogenesis assessment. Perfusion units were seen as indices of blood ﬂow. The blood ﬂow of the random-pattern skin ﬂaps was quantified by LDI Review software (version 6.1; Moor Instruments, Wilmington, DE, United States). Each mouse was measured three times and mean value was calculated.

### Hematoxylin and eosin staining

On postoperative day 7, six skin samples (1 cm × 1 cm) of the central area in ﬂap area II were acquired and sampled after sacrifice. The specimens were fixed in 4% paraformaldehyde for 24 h and embedded in paraffin for further transverse sectioning. Sections (5-mm thickness) were cut with a microtome and mounted on poly l-lysine–coated slides for H&E staining. Number of microvessels in per unit area (/mm^2^) were counted to assess the microvascular density (MVD) under a light microscope (×200 magnification; Olympus Corp., Tokyo, Japan).

### Immunohistochemistry

Six sections of the central part of area II in each group were deparaffinized in xylene and then rehydrated in a graded ethanol series. After washing, 3% hydrogen peroxide solution was added to the sections to block endogenous peroxidase. Then, 10.2 mM sodium citrate buffer (pH 6.0) was used for antigen retrieval (20 min, 95 °C). After blocking with 10% (w/v) bovine serum albumin for 1 h, the sections were incubated with primary antibodies: CD34 (1:100), VEGF (1:200), cadherin 5 (1 : 200), CASP3 (1 : 200), and SOD1 (1 : 100) overnight at 4 °C. After washing, the sections were further incubated with HRP-conjugated second antibody (1 : 1,000), stained with a DAB detection kit, and counterstained using hematoxylin. Slides were imaged at 200× magnification using the DP2-TWAN image-acquisition system (Olympus). Integral absorbance of VEGF-, cadherin 5-, CASP3-, SOD1- and CD34-positive blood vessels was calculated using Image-Pro Plus software (Media Cybernetics). Six random fields in three random sections of each tissue sample was counted.

### Western blotting

After euthanasia, skin samples (0.5 cm × 0.5 cm) from the middle of area II ﬂaps (*n* = 6) in each group were harvested and stored at −80 °C for western blotting analysis. After extracting the ﬂap tissues with lysis buffer, the protein concentration was measured using the BCA assay. An equal amount of 60 µg protein was separated by 10% (w/v) gel electrophoresis and transferred to polyvinylidene diﬂuoride membranes (Roche Applied Science, Indianapolis, IN, United States). After blocking with 5% (w/v) non-fat milk for 2 h at room temperature, the membranes were incubated with primary antibodies at 4 °C overnight: VEGF (1 : 1,000), MMP-9 (1 : 1,000), cadherin 5 (1 : 1,000), HO-1 (1 : 1,000), eNOS (1 : 1,000), SOD1 (1 : 1,000), Bax (1 : 1,000), CYC (1 : 1,000), caspase 3 (1 : 1,000), and GAPDH (1 : 1,000). After washing, the membranes were incubated with HRP-conjugated IgG secondary antibody (1 : 5,000) for 2 h at room temperature. The bands on the membranes were visualized using the ECL Plus Reagent Kit. Band intensity was quantified using Image Lab 3.0 software (Bio-Rad, Hercules, CA, United States).

### RT-PCR

Total RNA was extracted from skin samples *via* the RNA isolation kit (Accurate Biology, China) and the experimental manipulation was in strict accordance with manufacturer's instructions. Quantification was performed using a two-step reaction process: reverse transcription (RT) and PCR. Reverse transcription was performed in Evo M-MLV RT Master Mix Kit (Accurate Biology, China) for 15 min at 37 °C followed by 5 s at 85 °C. Reaction products (cDNA) were then diluted at suitable proportions and the diluted cDNA were used as template for PCR. Then, the real-time PCR was carried out *via* Pro Taq HS Probe Premix Kit (Accurate Biology, China). The reactions of RT-PCR were at 95 °C for 30 s, followed by 40 cycles of 95 °C for 5 s and 60 °C for 30 s. Every sample was analyzed in triplicate. Every Experiments were performed in triplicate.

### Statistics

Statistical analyses were performed using *GraphPad Prism* (Version 8.0.1, CA, United States). Data were presented as mean ± standard errors of the means (SEM). Statistical differences in comparison to the control group were analyzed using unpaired student *t*-tests. Significance was considered with *P* value < 0.05.

## Results

### Limonin elevated the survival of random skin flaps

**Figure 1:**
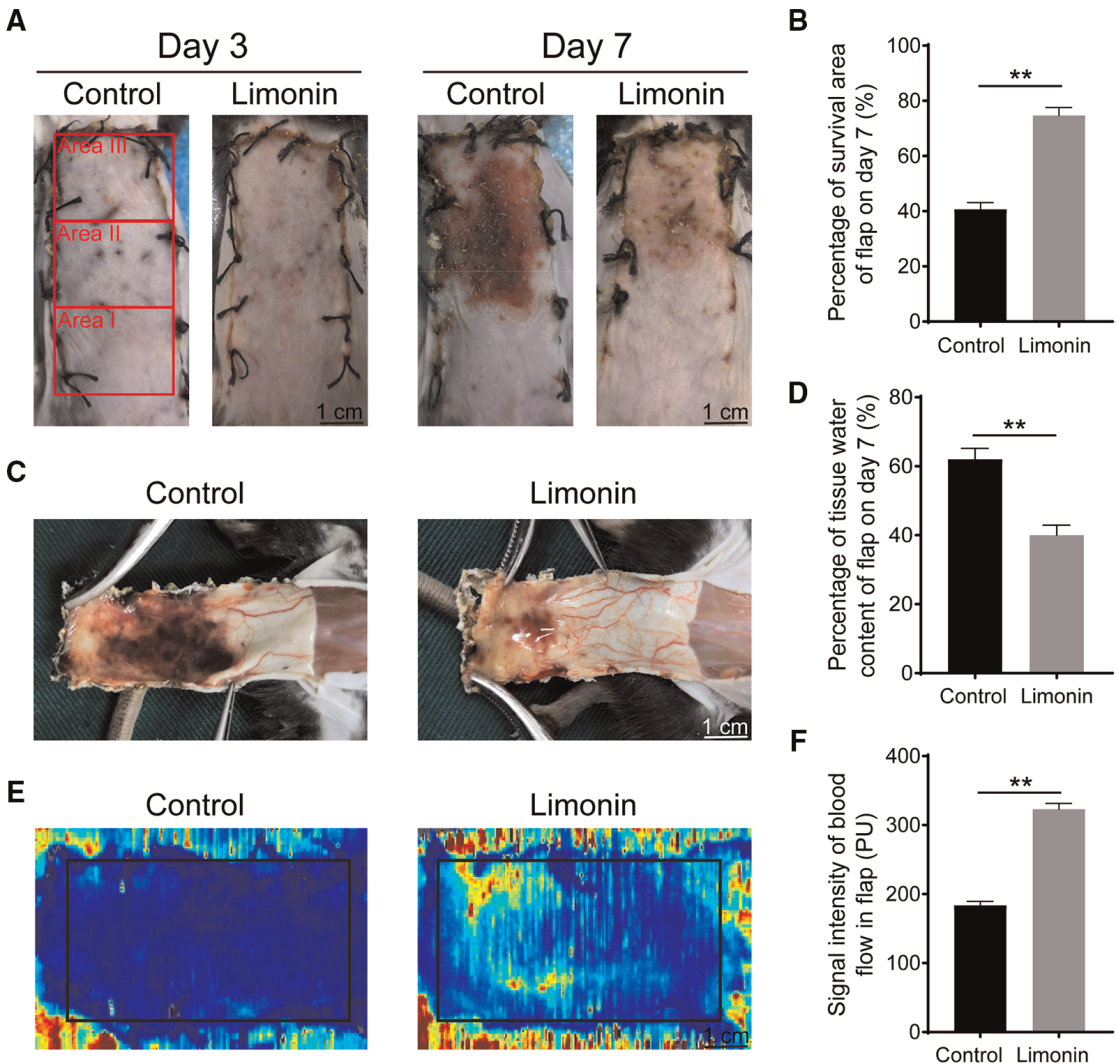
Limonin elevated the survival of random skin flaps. (**A**) Digital photographs of macroscopic surface of postoperative flaps on days 3 and 7 post-surgery (scale bar, 1 cm). (**B**) Histogram about percentage survival of random skin flaps on days 7 post-surgery. (**C**) Digital photographs of inner face of skin flaps, showing tissue edema and vessel statin of flaps. (**D**) Histogram about the percentage of tissue water content on days 7 post-surgery. (**E**) Visualization of blood flow below the tissue surface measured by LDBFI. (**F**) Histogram of blood flow on days 7 post-surgery. Data are mean ± standard error of the mean, *n* = 6 per group. **P < 0.01, vs. control group. LDBFI, Laser Doppler blood flow imaging.

On postoperative day 3, the appearance of the skin ﬂaps was pale and swollen in area III in both groups, without visible necrosis. No significant differences in ﬂap survival area were observed between the two groups (Figure 1A). On postoperative day 7, skin ﬂaps in area I exhibited survival in both groups, whereas the necrosis in area III had become darker and spread to area II, with scabbing and hardening (Figure 1A). The difference of survival area was more obvious than on day 3 between two groups, as the limonin group exhibited a significantly larger mean survival area than the control group (74.67 ± 2.93% and 40.67 ± 2.45%, respectively; *P* < 0.01; Figure 1B). To observe the extent of edema, ﬂap tissues were harvested from both groups to examine the distal portion of subcutaneous tissue. As shown in Figure 1C, the distal section of skin ﬂaps was swollen and bruised, with subcutaneous venous blood stasis in the control group, while these signs were less visible in the limonin group. After quantification, mean water content of flaps in the limonin group was much lower than that in the control group (40.00 ± 2.88% and 62 ± 3.19%, respectively; *P* < 0.01; Figure 1D), indicating that tissue edema was reduced after the limonin treatment. In addition, the limonin group showed more apparent blood ﬂow signal intensity in skin ﬂaps under the LDBF detection, than the control group (Figure 1E). After quantification, the blood ﬂow signal intensity was enhanced in the limonin group compared to the control group (323.00 ± 8.53 PU and 183.80 ± 5.71 PU, respectively; *P* < 0.01; Figure 1F).

### Limonin increased blood vessels in skin flaps

**Figure 2:**
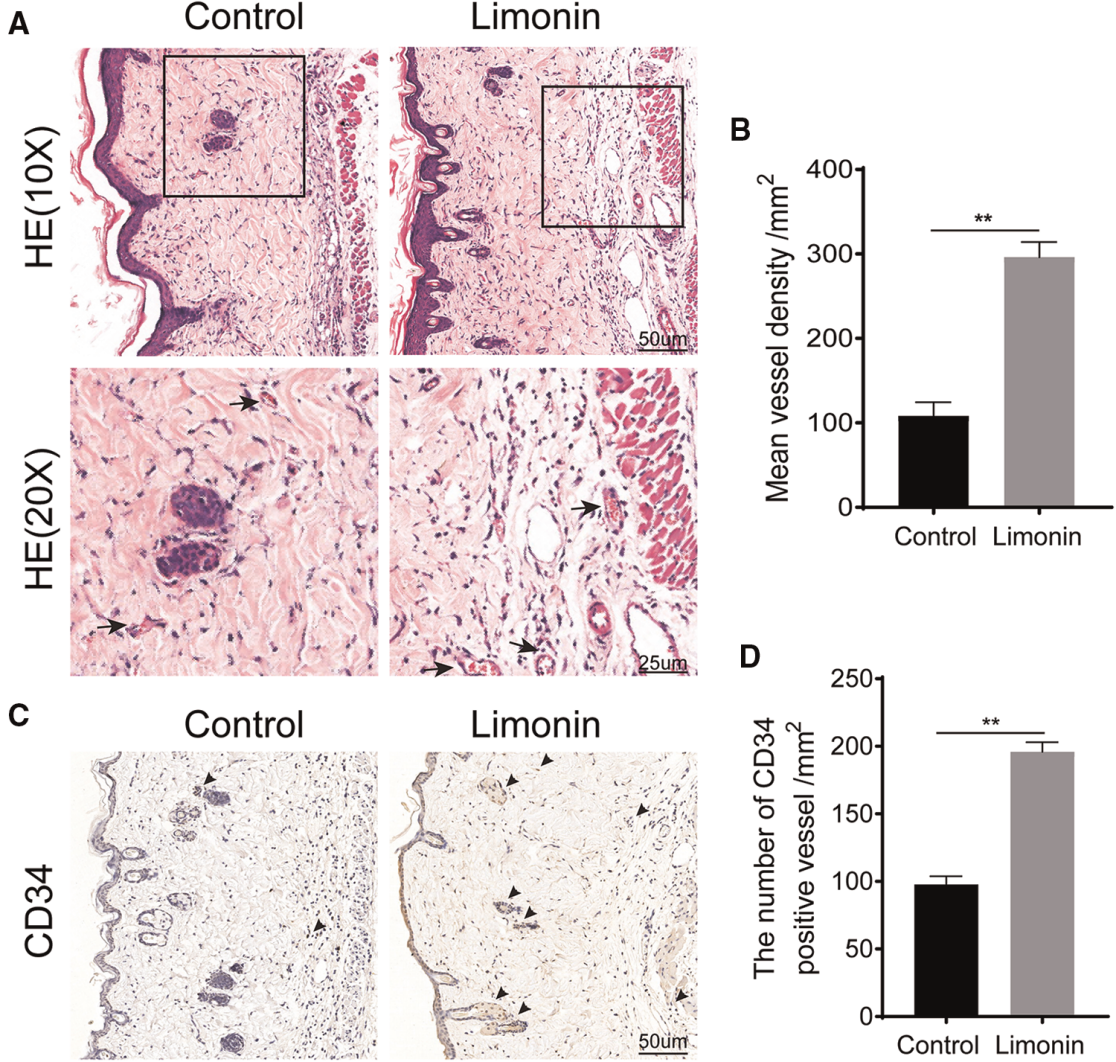
Limonin increased blood vessels in skin flaps. (**A**) H&E staining showing microvessels (located with black arrows) in ischemic skin flaps (area II) (original magnification ×200 and ×400; scan bar, 50 and 25 μm). (**B**) Mean vessel densities counted manually under H&E staining. (**C**) Immunohistochemistry staining of CD34-positive cells in flap area II. (**D**) CD34-positive vessel densities (/mm^2^) quantified under immunohistochemistry staining. Data are mean ± standard error of the mean, *n* = 6 per group. **P < 0.01, vs. control group.

Histological examination was performed to reveal neovascularization in ischemic skin flap between the two groups. As shown in Figure 2A, the limonin group exhibited more microvessels compared to the control group. We further calculated the mean MVD from the H&E staining, which reflected angiogenesis. The mean MVD in area II was significantly higher in the limonin group, than that in the control group (296.20 ± 7.33/mm^2^ and 108.20 ± 6.64/mm^2^; *P* < 0.01; Figures 2A,B). Immunohistochemistry of CD34 was performed to examine the endothelial cells in vessels of skin flaps. Immunohistochemistry revealed that the number of CD34-positive vessels increased in the limonin group, compared to the control group (196.00 ± 6.99/mm^2^ and 97.83 ± 5.95/mm^2^, respectively; *P* < 0.01; Figures 2C,D).

### Limonin promoted angiogenesis in skin flaps

**Figure 3:**
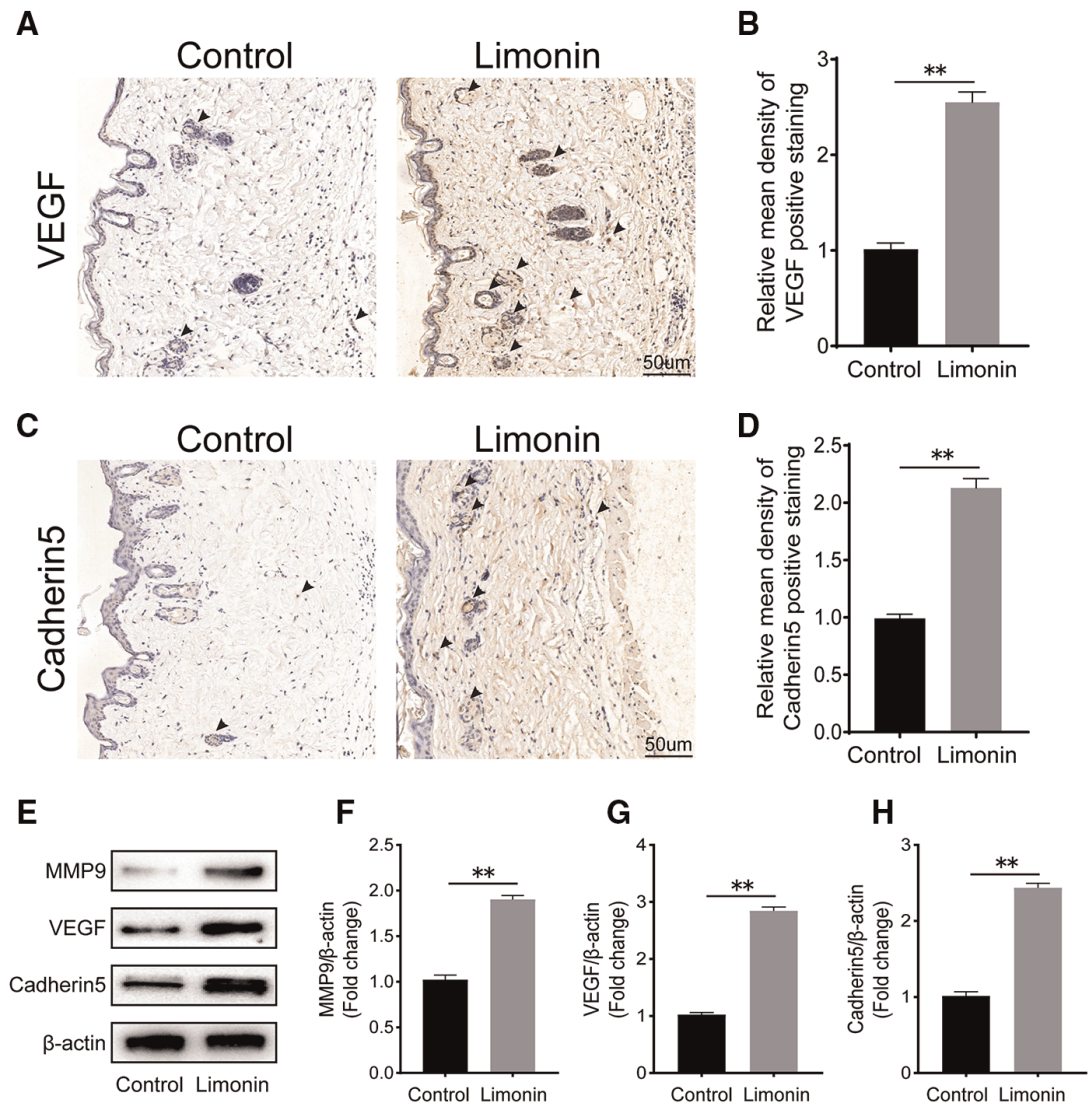
Limonin enhances angiogenesis in the ischemic area of the ﬂaps. (**A,C**) Immunohistochemistry for vascular endothelial growth factor (VEGF) and cadherin 5 expression in the ﬂaps of the control and limonin groups (original magnification ×200; scale bar, 50 µm). (**B,D**) Histograms of optical density values for VEGF and cadherin 5 by immunohistochemistry in each group. (**E**) Western blotting of the levels of VEGF, matrix metalloproteinase 9 (MMP9), and cadherin 5 in the control and limonin groups. Gels were electrophoresed under the same experimental conditions, and cropped blots are shown here. (**F–H**) Histograms of optical density values for MMP9, VEGF, and cadherin 5 as calculated by Western blotting. Data are mean ± standard error of the mean, *n* = 6 per group. *P < 0.05 and **P < 0.01, vs. control group.

To assess angiogenesis level in skin flaps, MMP9 and cadherin 5, vascular endothelial growth factor, were examined by immunohistochemistry. As depicted in Figure 3A, VEGF level in vessels and stromal cells was higher in the limonin group, compared to the control group (*P* < 0.01; Figure 3B). Similarly, Cadherin 5 was also significantly higher in the limonin group, compared to the control group (*P* < 0.01; Figures 3C,D). Furthermore, the expression of angiogenesis-related proteins, such as MMP9, VEGF, and cadherin 5, in area II of skin ﬂaps, was analyzed through western blotting (Figure 2E). Consistently, the optical density values of these proteins markedly increased in the limonin group than the control group (*P* < 0.01; Figures 3F–H).

### Limonin attenuated apoptosis and inflammation in skin flaps

**Figure 4:**
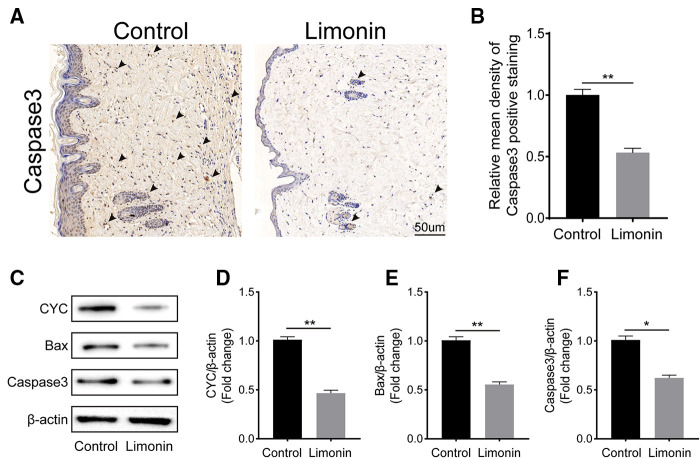
Limonin alleviates apoptosis in the ischemic area of the ﬂaps. (**A**) Caspase 3 levels of the ﬂaps in the control and limonin groups as assessed by immunohistochemistry (original magnification ×200; scale bar, 50 µm). (**B**) Histograms of optical density values for CASP3 by immunohistochemistry in each group. (**C**) Western blotting for Bax, CYC, and Caspase 3 expression in the skin ﬂaps of the control and limonin groups. Gels were electrophoresed under the same experimental conditions, and cropped blots are shown here. (**D–F**) Histograms of the optical density values of Bax, CYC, and Caspase 3 in the two groups as measured using Western blotting. Data are mean ± standard error of the mean, *n* = 6 per group. *P < 0.05 and **P < 0.01, vs. control group.

To examine cell apoptosis in the skin flaps, immunohistochemistry was performed to examine the level of Caspase 3 in the dermis layer of area II in the two groups. The Caspase 3 expression in vessels and stromal cells was lower in the limonin group, compared to the control group (Figure 4A), and so is the integral absorbance of Caspase 3 in the limonin group (*P* < 0.01; Figure 4B). In addition, western blotting was performed to detect the expression of Bax, CYC, and Caspase 3 in the skin ﬂaps (Figure 4C). The limonin group exhibited lower level of Bax, CYC, and Caspase 3 than that in the control group (*P* < 0.01, *P* < 0.01, and *P* = 0.018, respectively; Figures 4D–F).

To evaluate the anti-inflammatory effects of the Limonin in the skin flaps modle, we utilized RT-PCR to examine the level of iNOS, IL-6 and IL-1β in the dermis layer of area II in the two groups. As has typically been reported in the literature, Limonin could inhibit inflammation gene compared with control group *in vivo* (Supplementary Figure S1A).

### Limonin ameliorated the oxidative stress in skin flap

**Figure 5:**
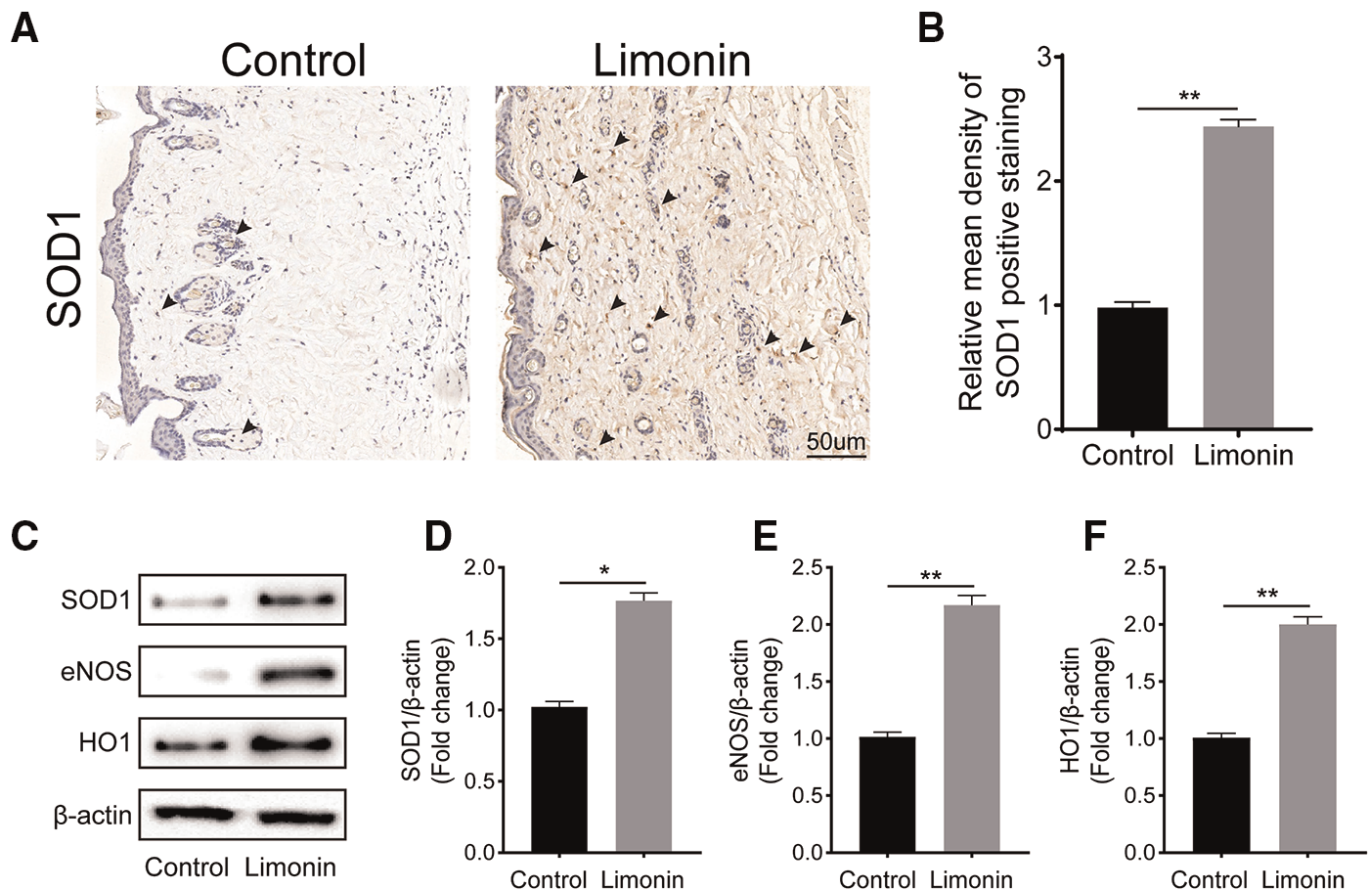
Limonin attenuates oxidative stress in the ischemic area of the ﬂaps. (**A**) Immunohistochemistry for superoxide dismutase 1 (SOD1) expression in the ﬂaps of each group (original magnification ×200; scale bar, 50 µm). (**B**) Histograms of optical density values of SOD1 by immunohistochemistry in each group. (**C**) Western blotting for SOD1, endothelial nitric oxide synthase (eNOS), and heme oxidase 1 (HO1) protein expression in the skin ﬂaps. Gels were electrophoresed under the same experimental conditions, and cropped blots are shown here. (**D–F**) Histograms of the optical density values of SOD1, eNOS, and HO1 in the two groups as evaluated by Western blotting. Data are means ± standard error of the mean, *n* = 6 per group. *P < 0.05 and **P < 0.01, vs. control group.

To verify the effects of limonin on oxidative stress in the skin ﬂaps, immunohistochemistry was performed to determine SOD1 level, which reﬂected the magnitude of the oxidative stress in area II of the skin ﬂaps in two groups. As shown in Figure 5A, the level of SOD1 and its integral absorbance were higher in the dermis in the limonin group, compared to the control group (*P* < 0.01; Figure 5B). Moreover, western blotting was performed to detect the expression of SOD1, eNOS, and HO1 in the skin ﬂaps (Figure 5C). The optical density values of these proteins were obviously upregulated in the limonin group than that in the control group (*P* = 0.032, *P* < 0.01, and *P* < 0.01, respectively; Figures 5D–F).

## Discussion

In our current study, limonin is demonstrated to be a novel therapeutic strategy for the treatment of ischemic random skin flap. After the operation of skin flap, limonin treatment may be able to promote improved angiogenesis, reduced apoptosis, decreased oxidative stress, and increased survival of random skin flap.

Ischemic necrosis in distal portion of random skin flap is a common and influential complication (2). The confluence of postoperative ischemia and subsequent ischemia reperfusion injury can further induce necrosis of skin flaps. As previous reported, damage by free oxygen radials and cell apoptosis played an important role in the pathology of flap necrosis (16). Based on previous researches, agents with the effect of antioxidant, anti-apoptosis, and pro-angiogenesis may be able to promote random skin flap survival. Accumulated evidence showed that limonin possessed the effect of anti-inflammatory and antioxidant, however, few studies have explored the therapeutic effect of limonin for random skin flap survival. Thus, the current study demonstrated that limonin significantly decreased ischemic necrosis in skin flaps.

After the operation, skin flaps undergoes plasma exudation, congestion and edema, and ischemia. After that, skin flap initiates the angiogenesis process and new blood flow is introduced, alleviating the ischemia and necrosis of skin flaps (17). According to the previous researches, limonin was strongly involved in the ischemia related diseases (11, 18). Xiong et al. have demonstrated that limonin was a promising target for coronary ischemia of myocardial infarction. In the present study, we found that higher mean vessel density and CD34 expression were detected after limonin treatment, consistent with higher blood flow according to LDBFI examination. Thus, we hypothesize that limonin treatment is likely to improve the vascularization and reduce necrosis in ischemic random skin flap. In the process of angiogenesis, MMP9 is a crucial factor in the destruction of preexisting cell connections, and VEGF plays a critical role in mitosis of endothelial cells and Cadherin5 helps to form intercellular junctions which is conducive to maturation of new capillaries (19–21). Therefore, we examined the expression levels of the three factors in the skin flap. Immunohistochemistry analysis showed that limonin significantly elevated the levels of VEGF and Cadherin5 in ischemic flaps. Moreover, western blotting also indicated that higher expressions of MMP9, VEGF, and Cadherin5 were detected in the random skin flap after limonin treatment. Taken together, limonin is effective to promote angiogenesis and reduces necrosis in ischemic skin flaps with elevated level of MMP9, VEGF, and Cadherin5.

It is well-known that skin flap survival is easily damaged by ischemic reperfusion injury after the flaps operation (22). In the ischemic skin flaps, angiogenesis and subsequent blood supply not only bring oxygen, but also lead to the production of oxygen free radical, contributing to damage of oxidative stress (23). During the oxidative stress process, oxygen free radical acts on lipid peroxidation in cell membrane and further invalidates related proteins (24, 25). Malondialdehyde is a reaction product of lipid peroxidation, which is proportional to the level of oxidative stress (26). At the same time, several antioxidant substances are synthesized and secreted to lower the level of oxidative stress during the process, including GSH and SOD (27). eNOS and HO1 are two important enzymes involved in the antioxidant activity. Li et al. have indicated that limonin was effective to alleviate non-alcoholic fatty liver disease through reducing lipid accumulation, inhibiting inflammation and oxidative stress, and the mechanism may depend on NRF2/HO-1 signaling pathway (28). In the current study, we have also demonstrated that limonin was able to increase the expression of SOD1, eNOS, and HO1 in ischemic skin flaps through immunohistochemistry and western blotting analysis. These results in our study reveal that limonin obviously inhibits oxidative stress in random skin flaps. Anti-inflammation may be effective for skin flap survival while limonin has been reported to exert anti-inflammation effect. The anti-inflammation effect of limomin was explored in our experiments. These results suggest that limonin may exert an anti-inflammatory effect in random skin flaps.

Furthermore, limonin treatment showed less apoptosis of endothelial cells and decreased permeability, leading to less edema. Consistent with our result, Yang et al. have demonstrated that limonin could inhibit APAP-induced mitochondrial-mediated apoptosis by decreasing the ratio of Bax/Bcl-2, recovery of mitochondrial membrane potential (MMP), inhibiting ROS production and cleavage of caspase-3 in L-02 cells (29). The apoptosis process was detected with impairment in vascular endothelial cells and basal layer. The injury of vascular endothelium might cause plasmatic permeability, obvious congestion and edema, ischemia and necrosis (17). During the process of apoptosis, increased permeability of mitochondrial outer membranes may lead to the mitochondrial swelling, which is partly induced by Bax (30, 31). Moreover, CYC is released from the mitochondria, participating in the formation of apoptosome (31). Then Caspase 3 was activated *via* a cascade reaction, as a crucial apoptosis executor, Caspase 3 indicates the level of apoptosis in tissues (32). In our study, we examined the expression of Bax, CYC, Caspase 3 through immunohistochemistry and western blotting analysis. Results showed that limonin inhibited the activation of apoptosis in random skin flaps. Therefore, our results suggest that limonin could decrease apoptosis through reducing the expression of Bax, CYC, and Caspase 3, leading to less necrosis of random skin flaps.

There are also some limitations existed in our study. The possibility of long-term effect of limonin treatment has not been assessed. Optimal drug dose, timing, and duration of management also need to be explored comprehensively through well-designed prospective randomized controlled trials in the future. Examination of MMP9, VEGF, and Cadherin5 are the result of angiogenesis, although these gene expressions may indicate the effect of angiogenesis to some extent, the exact effect of angiogenesis still needs to explored. Moreover, gene expression should be verified in our further experiments. Nevertheless, results from present studies suggest that limonin is a simple, safe, effective, and economical management to decrease skin flap necrosis.

## Conclusion

Limonin is effective to enhance survival of skin flaps through promoting angiogenesis, reducing oxidative stress and inhibiting apoptosis. Thus, the present work provided a potent therapy for the survival of random skin ﬂaps. The actual role of limonin in human skin ﬂap transplantation needs further experimental verification.

## Data Availability

The raw data supporting the conclusions of this article will be made available by the authors, without undue reservation.
