# Supplementary figures and images for: Effects of limonin treatment on the survival of random skin flaps in mice
Source: Front Surg. 2023 Jan 6;9:1043239. doi: 10.3389/fsurg.2022.1043239 (PMC9852612; doi:10.3389/fsurg.2022.1043239)

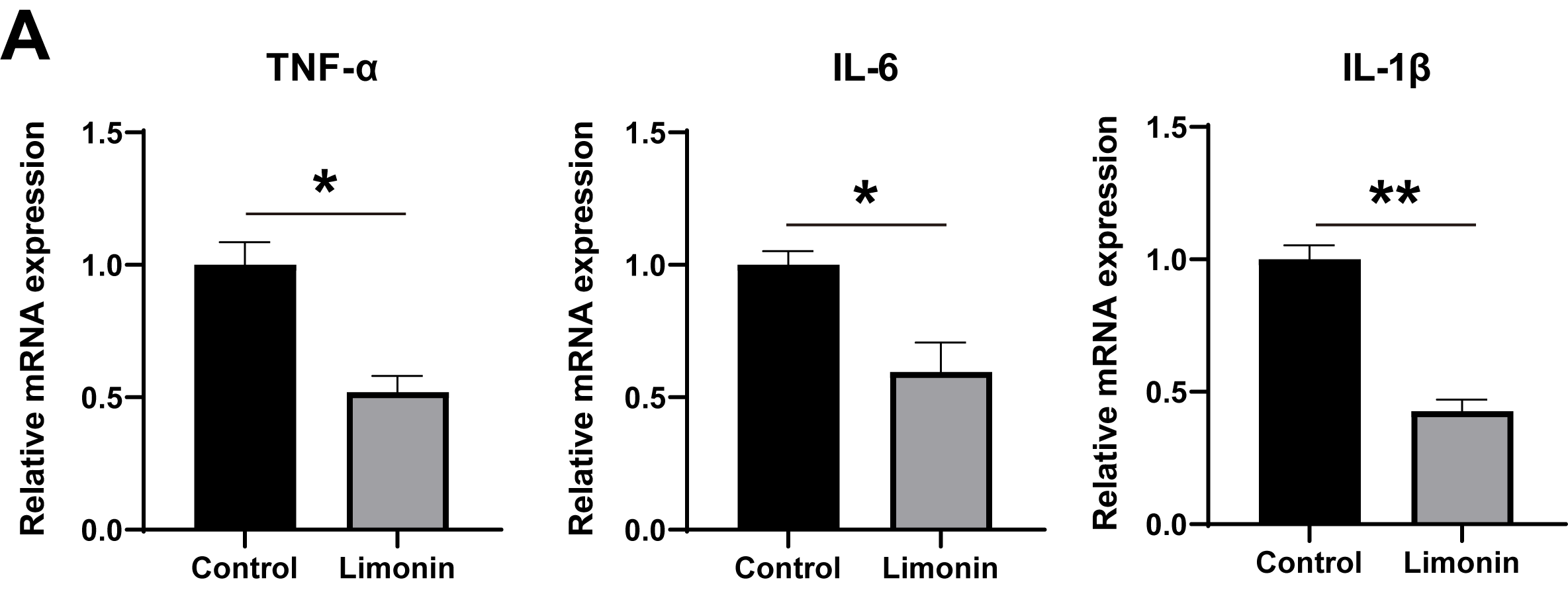

Supplement: Supplementary file 1 [file FigureS1.tif]
